# Status of cardiovascular health in United States immigrants using the American Heart Association's Life's Essential 8 framework: a population-based study of prevalence estimates and determinants

**DOI:** 10.1016/j.lana.2025.101107

**Published:** 2025-05-13

**Authors:** Nour Makarem, Rahul Hosalli, Ariana Lopez, Vanessa Dinh, Billy A. Caceres, Amanda C. McClain, Pricila H. Mullachery, Tala Al-Rousan, Odayme Quesada

**Affiliations:** a Department of Epidemiology, Mailman School of Public Health, Columbia University Irving Medical Center, 722 West 168th St., New York, NY, 10032, USA; b Center for Research on People of Color, Columbia University School of Nursing, 560 West 168th St., New York, NY, 10032, USA; c School of Exercise and Nutritional Sciences, San Diego State University, 5499 Aztec Bowl., San Diego, CA, 92182, USA; d Department of Health Services Administration and Policy, College of Public Health, Temple University, 1301 Cecil B. Moore Ave., Philadelphia, PA, 19122, USA; e Herbert Wertheim School of Public Health and Human Longevity, University of California, San Diego, 9500 Gilman Dr, La Jolla, CA, 92093, USA; f Women's Heart Center, The Christ Hospital Heart and Vascular Institute, 5885 Harrison Ave #1900, Cincinnati, OH, 45248, USA; g Department of Internal Medicine, University of Cincinnati College of Medicine, 231 Albert Sabin Way #45229, Cincinnati, OH, 45229, USA

**Keywords:** Cardiovascular health, Life's Essential 8, Immigrant health, Social determinants of health, Health equity

## Abstract

**Background:**

The United States (US) has the largest immigrant population globally. Immigrants endure social and structural factors that adversely influence cardiovascular health (CVH), a construct focused on health preservation, not merely the absence of disease, and defined by eight modifiable health factors and behaviors that support healthy longevity. We compared overall and individual CVH metrics between immigrants and non-immigrants and characterized CVH and its determinants among immigrant sub-populations.

**Methods:**

The analytic sample included 13,471 adults (19% immigrants, i.e., foreign-born, 51% female), ages 20–79 y (mean ± SD for immigrants and non-immigrants: 44.8 ± 4.8 y and 45.7 ± 11.2 y, respectively) from the 2013 to 2018 National Health and Nutrition Examination Survey. CVH was characterized consistent with the American Heart Association's (AHA) Life's Essential 8 (LE8) guidelines (metrics: body mass index (BMI), blood glucose, blood lipids, blood pressure, nicotine use, sleep health, diet quality, and physical activity; score range: 0–100, low CVH: LE8 score <50). CVH scores were compared among immigrants and non-immigrants and by sex, ethnicity, years in the US, and citizenship among immigrants. Survey-weighted regression models evaluated psychosocial and demographic factors in relation to CVH.

**Findings:**

Immigrants had higher overall CVH scores (69.1 vs. 66.4) (p < 0.0001) and significantly higher subscores for diet (52.5 vs. 38.8), nicotine exposure (80.3 vs. 68.0), BMI (61.6 vs. 57.1), and blood pressure (74.1 vs. 71.8), but lower physical activity (47.0 vs. 52.6), glucose (82.2 vs. 86.1), and cholesterol (63.0 vs. 68.5) scores compared to non-immigrants. Among immigrants, those who were male vs. female (67.5 vs. 70.7) (p = 0.003), Hispanic vs. non-Hispanic (66.6 vs. 71.5) (p < 0.0001), and living in the US ≥15 y vs. <15 y (67.7 vs. 71.8) (p < 0.0001) had lower CVH. In regression models, being male, 45+ y, or Hispanic, having food insecurity, lower education and income, depression, no health insurance, and ≥15 y living in the US were associated with lower CVH.

**Interpretation:**

While US immigrants have more favorable overall CVH compared to US-born persons, CVH status is complex and heterogenous across immigrant sub-populations. Glycemic control, physical inactivity, and blood lipids may be important targets for CVH promotion interventions in this population; Hispanic immigrants and those who lived in the US for ≥15 y may represent key subpopulations to engage in these efforts.

**Funding:**

10.13039/100000002National Institutes of Health and 10.13039/100000968American Heart Association.

**Research in context**

**Evidence before this study**

The United States (US) has the largest immigrant population globally accounting for one fifth of the global migrant population. US immigrants are highly diverse, representing every country around the world, and the US immigrant population is estimated to reach ∼80 million by 2065. Immigrants endure social and structural factors that could adversely impact their cardiovascular health (CVH). We searched PubMed and Google Scholar up to March 12, 2025 with no limits on publication dates or language, for papers on cardiovascular risk factors in immigrant populations using the search terms “cardiovascular health”, “cardiovascular disease risk factors”, “immigrants”, “foreign-born individuals”. While previous studies show that cardiovascular disease risk varies substantially between immigrants and their host populations, there are no studies on CVH, defined by the American Heart Association's Life's Essential 8 framework, in the US immigrant population; existing studies evaluate some cardiovascular risk factors, focus primarily on US Hispanic immigrants, and have not been conducted within nationally representative cohorts.

**Added value of this study**

Since the publication of the American Heart Association's Life's Essential 8, representing an enhanced framework for the assessment of CVH at the population level that is strongly linked to CVD, chronic disease, aging and mortality outcomes, the status of CVH has been examined in the general US population but not among the immigrant population. Using data from a nationally representative cohort, we show that US immigrants generally had better overall CVH compared to non-immigrants, but immigrants had poorer glycemic and blood lipid profiles and lower physical activity levels compared to non-immigrants. Among US immigrants, those who were male, Hispanic, and living in the US for 15 or more years had lower CVH. Household food insecurity, lower education and income levels, lack of health insurance, and poorer psychological health were determinants of lower CVH in this population.

**Implications of all the available evidence**

CVH status is complex and varies by CVH metric, including across immigrant sub-populations. Our findings are necessary to support evidence-based decisions in healthcare policy among immigrants and highlight that future resource allocation and primordial prevention efforts in this population should focus on glycemic and blood lipids control and improving physical activity levels, with Hispanic immigrants and immigrants with more years in the US being key subpopulations to engage in CVH promotion efforts. Interventions aimed at preserving healthy lifestyle habits among new immigrants and addressing socioeconomic inequities and mental health could also have great impact. Given the growing number of individuals affected by forced displacement due to climate change, political conflict, and violence, the influence of the root causes driving immigration on CVH warrants further investigation.

## Introduction

The United States (US) has the largest immigrant population globally, with a record high ∼47 million foreign-born residents in 2022 accounting for one fifth of the world's migrant population.^1^^,^^2^ US immigrants are heterogenous, representing every country around the world, with about half of the foreign-born population being Hispanic.^2^ If current demographic trends persist, the US immigrant population is estimated to reach ∼80 million by 2065 with most coming from Latin America and Asia and approximately a quarter of immigrants coming from other regions such as Europe, Canada, the Middle East and North Africa and Sub-Saharan Africa.^2^^,^^3^ Increasingly, more immigrants are entering the US as refugees and asylum seekers who tend to have a higher risk of cardiovascular disease (CVD) compared to other immigrants and host communities.^4^ Immigrants endure social and structural factors that may adversely influence their cardiovascular health (CVH) and contribute to CVH disparities; this becomes more pronounced the longer immigrants reside in host countries, such as the US, due to acculturation, cumulative exposure to these factors, and experiencing discrimination, structural racism, and anti-immigrant rhetoric.^1^^,^^5^^,^^6^

Immigrants and their host populations differ substantially in CVD morbidity and mortality.^6^, ^7^, ^8^ Given the strong association between CVH and future risk of CVD,^9^^,^^10^ disparities in CVH between US immigrants and non-immigrants likely underlie these differences in CVD outcomes. Despite being more socioeconomically disadvantaged and facing structural and institutional inequities in the host society, immigrants are paradoxically often shown to be healthier than the host population.^1^^,^^11^ However, this health advantage diminishes over time due to assimilation of unhealthy lifestyles and longer exposure to institutional and societal inequities.^1^^,^^11^ While some prior studies have examined immigration as a social determinant of health (SDOH) in the context of CVD prevention,^1^^,^^12^ there are no prior studies that have investigated differences in CVH between US immigrants and non-immigrants using the American Heart Associations (AHA) Life's Essential 8 (LE8) framework.^13^ In addition, the contribution of demographic factors, psychological health, and SDOH, including socioeconomic factors and healthcare access, to CVH status as defined by the LE8 has not been investigated in the US immigrant population. Understanding the drivers behind CVH inequities may help to mitigate the unequal burden of CVD and identify new avenues for intervention in the population at large.

The purpose of this paper is to describe current differences in prevalence and distributions of CVH between US immigrants, defined by nativity status (i.e., being born outside of the US),^14^ and non-immigrants using the AHA's LE8 framework and data from the 2013 to 2018 National Health and Nutrition Examination Survey (NHANES), characterize differences in overall and individual metrics of CVH among immigrant subgroups, and elucidate determinants of poor CVH in the US immigrant population.

## Methods

### Study population

Participants were 13,471 adults, aged 20–79 years, from the 2013 to 2018 NHANES, and constitute a representative sample of the civilian, non-institutionalized US population selected through a complex, multistage probability sampling design.^15^ Participants provided written informed consent and completed home interviews and mobile examinations to provide socio-demographic, lifestyle, psychological status, anthropometric, and physiologic data. NHANES data and guidance on analytical approaches are available from the US Centers for Disease Control and Prevention's National Center for Health Statistics and can be accessed at: https://www.cdc.gov/nchs/nhanes/index.htm. NHANES is approved by the National Center for Health Statistics Ethics Review Board. This research is exempt by the Columbia University Irving Medical Center Institutional Review Board given the de-identified nature of the data. The total combined sample of the 2013–2018 NHANES was comprised of 29,400 participants. For this analysis, individuals with an incomplete exam or survey (n = 1339), aged <20 years or >79 years (n = 12,791), who were pregnant or breastfeeding (n = 314), diagnosed with CVD (i.e., heart failure, coronary heart disease, angina, heart attack, and stroke) (n = 1436) or without a known immigration status (n = 49) were excluded. Consistent with the definition proposed by *The Lancet* Commission on Migration and Health,^14^ we define an immigrant as any person who has moved away from their place of birth and habitual residence into a different country. Participants with existing CVD were excluded, given the focus of the LE8 guidelines on primordial prevention and prevention of the onset of CVD, and consistent with the methodology used by Lloyd–Jones et al. in the AHA Presidential Advisory's accompanying publication describing the status of CVH in the US general population.^16^ Our final analytic dataset was comprised of 13,471 adult participants (Supplementary Figure S1).

### Assessment and quantification of cardiovascular health

The operationalization of the LE8 scoring algorithm is described in detail in our prior publication by Dinh et al.^17^ and consistent with the AHA Presidential Advisory on Life's Essential 8 and the accompanying publication on the status of CVH in the US population using NHANES.^13^^,^^16^ Briefly, the LE8 CVH score is composed of four health factors (body mass index (BMI), blood glucose, blood lipids, blood pressure) and four health behaviors (nicotine use, sleep health, diet quality, and physical activity).

Health factors were assessed at the mobile examination, and health behaviors were self-reported in the interview portion of NHANES as described previously.^17^ Blood pressure was measured by a physician certified in blood pressure measurement. Three measurements were obtained using a sphygmomanometer with the first measurement occurring after the individual had rested for 5 min and the two additional measurements being obtained 30 s apart using the same arm. The mean of the three blood pressure measurements was used. BMI was calculated using anthropometric measures (body weight (kg)/height (m^2^)). Blood samples were also obtained to measure total cholesterol, high-density lipoprotein (HDL) cholesterol, fasting blood glucose and/or glycosylated hemoglobin (HbA1c). Participants also self-reported blood pressure, diabetes, and cholesterol-lowering medications use, which were considered for scoring the blood pressure, blood glucose, and blood lipids metrics, respectively.

Diet was ascertained from two 24-h recalls and was used to evaluate the adherence to a Dietary Approaches to Stop Hypertension style eating pattern, with scores ranging from 0 to 100 and higher scores indicative of higher Dietary Approaches to Stop Hypertension style diet adherence.^13^ The minutes per week of moderate to vigorous physical activity were estimated using data on the frequency and duration of recreational physical activity (e.g., exercise and sports). Smoking status was determined using self-reported information from the family household questionnaire regarding lifetime smoking habits, current smoking habits, years since quitting, and exposure to secondhand smoke. Sleep health was measured by habitual sleep duration (in hours) computed as the weighted mean of self-reported sleep duration on weekdays and weekends.

Consistent with the recommended LE8 scoring algorithm,^13^ individual CVH metric scores were computed and ranged from 0 to 100 such that higher scores reflected greater concordance to each LE8 guideline. Detailed scoring for LE8 metrics is shown in Supplementary Table S1. The overall LE8 score was computed as the unweighted mean of the eight component scores and also ranged from 0 to 100 with higher LE8 scores representing better overall CVH. Per the LE8 guidelines,^13^ an overall LE8 score of 80–100 was considered indicative of high CVH, while LE8 scores of 50–79 and 0–49 were considered indicative of moderate and low CVH, respectively.

### Assessment of covariates

Sociodemographic characteristics and SDOH including age, sex, ethnicity, marital status, educational attainment, household income, health insurance status and type of health insurance, home ownership status, household food insecurity, citizenship status, and number of years living in the US were all self-reported during the interviews. The number of years living in the US was used as a proxy for acculturation level consistent with prior work.^11^ Citizenship status was ascertained using the response to the question “Are you a citizen of the United States?”. In NHANES, a poverty level index was computed as the ratio of self-reported family income to poverty thresholds specific to family size defined by the Department of Health and Human Services, and the index was then grouped into two categories >1.30 and ≤1.30 consistent with commonly used percentages of the poverty guidelines (i.e., 130% of the guidelines), by federal programs, in determining eligibility.^15^ Consistent with prior research on CVH in the US population,^16^^,^^17^ depression was used as a proxy for psychological heath, given that it was consistently assessed across NHANES cycles. Depression was measured using the Patient Health Questionnaire-2 (PHQ-2), which asks participants about the frequency of depression symptoms with scores ranging from 0 to 6 and a score ≥3 indicating a probable case of depression.^18^ Household food security status was measured using the 18-item US Household Food Security Survey Module.^19^ Affirmative responses were tallied (range: 0–18) and households were classified as food secure (score 0–2) or food insecure (3–18) based on United States Department of Agriculture guidelines.^19^

### Statistical analysis

The guidelines provided by NHANES were adhered to for combining data across cycles and accounting for the complex multistage sampling design in all analyses.^15^ T-test and Chi-square tests were used to compare sociodemographic characteristics between immigrants and non-immigrants. Student's t-tests were used to compare mean CVH scores by nativity (immigrant (i.e., foreign-born) vs. non-immigrant (i.e., US-born)), and among immigrants, by sex (male vs. female), ethnicity (Hispanic vs. non-Hispanic), years in the US (≥15 years vs. <15 years), and citizenship status (US citizen vs. non-citizen). Survey-weighted linear models evaluated sociodemographic and psychological health factors as well as SDOH in relation to the LE8 score (continuous), while survey-weighted logistic models were used to estimate odds ratios (OR) (95% confidence intervals (CI)) representing the ratio between the odds of having moderate to high CVH (LE8 score of 50–100) and the odds of having poor CVH (LE8 score of 0–49). In logistic models, all independent variables were dichotomized as follows: age (20–44 vs. 45+ y), sex (Male vs. Female), ethnicity (Hispanic vs. non-Hispanic), marital status (Married/Living with partner vs. Single/Widowed/Divorced), educational attainment (Some college and above vs. Less than college), household income (≥$45,000 vs. <$45,000), poverty index (>1.30 vs. ≤1.30), health insurance (Yes vs. No), private insurance (Yes vs. No), home ownership (Yes vs. No), household food security status (High/Marginal vs. Low/Very Low), depression (PHQ-2 ≥3 vs. PHQ-2 <3), years in the US (≥15 years vs. <15 years vs.), and citizenship (Citizen vs. Non-Citizen). In linear models, independent variables were also dichotomized, with the exception of age, PHQ-2 scores and the Poverty Index. In exploratory analyses, we also conducted linear and logistic regression models comparing LE8 scores and CVH status of Hispanic immigrants, the largest US immigrant group, to non-Hispanic Asian immigrants, the second largest US immigrant group. For all presented statistical analyses, we conducted a complete case analysis. Overall, most variables had a missingness rate <10% (vast majority had missingness rates <5%) among immigrants, with the exception of diet (13%), poverty index (14%), depression (12.5%), and private insurance (27%). Analyses were performed using SAS 9.4 (SAS Institute) and p-values <0.05 were considered statistically significant.

### Role of the funding source

The funders had no role in study design, data collection, data analysis, interpretation, or writing of this report.

## Results

### Characteristics of study population by self-reported immigration status

The characteristics of the analytical sample (*n* = 13,471) are shown in Table 1. Females comprised about half of the sample and those aged 40–64 years were the largest age group in both immigrant and non-immigrant populations. Immigrants self-identified predominately as Mexican (27%), non-Hispanic Asian (27%), and other Hispanic (22%). Non-immigrants were predominately non-Hispanic White (75%) and non-Hispanic Black (13%) (p < 0.0001). Immigrants were more likely to be married or living with a partner (72% vs. 62%, p < 0.0001). In terms of socioeconomic status, immigrants were less likely to have a college education and more likely to have a family income <$45,000; the prevalence of poverty was higher among immigrants with about a third of immigrants having a poverty index ≤1.30 compared to less than one fifth of US-born individuals (p < 0.0001). In addition, compared to non-immigrants, immigrants were less likely to have health insurance (70% vs. 87%), particularly private insurance (61% vs. 84%), to own a home (54% vs. 69%), and to be food secure (78% vs. 84%) (p < 0.0001 for all).

**Table 1:** Characteristics of United States adults by self-reported immigration status; National Health and Nutrition Examination Survey, 2013–2018.^a^

	Immigrant^b^	Non-immigrant	p-value
	Prevalence, % (N, weighted)	Prevalence, % (N, weighted)	
**Total sample n** = **13,471, weighted to 193,408,360 adults**	19.0 (36,808,294) N = 4503	81.0 (156,600,066) N = 8968	
**Mean age, years (SD)**	44.8 (4.8)	45.7 (11.2)	0.014
**Age, years**			
20–39	39.9 (14,671,053)	38.8 (60,712,287)	<0.0001
40–64	49.4 (18,196,571)	46.9 (73,468,165)	
65–79	10.7 (3,940,670)	14.3 (22,419,615)	
**Sex**			
Male	49.6 (18,258,750)	48.5 (75,911,647)	0.28
Female	50.4 (18,549,543)	51.5 (80,688,419)	
**Self-reported race and ethnicity**			
Non-Hispanic Asian	26.8 (9,862,387)	0.9 (1,486,944)	<0.0001
Non-Hispanic Black	6.9 (2,551,162)	12.6 (19,727,574)	
Non-Hispanic White	14.6 (5,372,431)	74.5 (116,709,119)	
Mexican	27.2 (10,007,009)	5.1 (8,036,262)	
Other Hispanic	21.5 (7,931,633)	3.0 (4,745,769)	
Other race, including multi-racial	2.9 (1,083,671)	3.8 (5,894,397)	
**Marital status**			
Single/widowed/divorced	28.5 (10,495,109)	37.9 (59,265,375)	<0.0001
Married/living with partner	71.5 (26,305,948)	62.1 (97,309,472)	
**Educational attainment**			0.010
High school/GED and below	48.6 (17,856,318)	32.3 (50,630,228)	
Some college and above	51.4 (18,915,274)	67.7 (105,944,456)	
**Household income**			<0.0001
<$45,000	46.8 (15,572,624)	35.3 (53,102,234)	
≥$45,000	53.2 (17,720,225)	64.7 (97,361,262)	
**Poverty index**^c^			
≤1.30	33.2 (10,539,897)	18.4 (26,885,458)	<0.0001
1.31–1.85	13.4 (4,262,066)	9.5 (13,919,051)	
1.86–3.50	22.8 (7,240,775)	24.6 (35,979,239)	
>3.50	30.5 (9,692,976)	47.4 (69,330,677)	
**Health insurance**			<0.0001
No	30.0 (11,027,303)	12.8 (20,006,213)	
Yes	70.0 (25,695,605)	87.2 (136,426,218)	
**Private insurance**			<0.0001
No	38.8 (11,027,303)	16.4 (20,006,213)	
Yes	61.2 (17,358,177)	83.6 (102,048,366)	
**Home ownership**			<0.0001
No (rented or other arrangement)	45.9 (16,267,993)	31.2 (47,862,013)	
Yes (owns home)	54.1 (19,195,054)	68.8 (105,310,258)	
**Household food security**			0.010
Low/very low	22.0 (7,825,175)	15.7 (24,002,804)	
High/marginal	78.0 (27,664,584)	84.3 (129,240,629)	
**Depression**^d^			0.80
Not a probable case	93.4 (36,114,049)	92.4 (150,235,195)	
Probable case	6.6 (2,560,045)	7.6 (12,309,521)	
**Years in US**			
<15 years	38.8 (13,593,669)	–	
≥15 years	61.2 (21,408,102)	–	
**Citizenship**			
Non-citizen	50.8 (18,685,839)	–	
Citizen	49.2 (18,122,454)	–	

GED, General Education Development; PHQ, Patient Health Questionnaire.

a NHANES data from 2013 to 2018 cycles were combined. The NHANES analytical guidelines for combining data across cycles were used to construct sample weights. These were used to estimate the number of individuals in the United States population overall and in each group. T-tests and Chi-square tests were used to compare characteristics between immigrants and non-immigrants. While the total sample size and sample size of immigrant and non-immigrant populations is shown, all descriptive and analytical estimates consider NHANES analytical guidelines and sample weights to ensure that findings representative and generalizable to the broader United States population.

b We define an immigrant as any person who has moved away from their place of birth and habitual residence into a different country. Therefore, participants who were born outside of the United States were considered immigrants.

c The poverty index is the ratio of family income to poverty. Poverty guidelines are set by the Department of Health and Human Services and are issued each year for determining financial eligibility for certain federal programs.

d The PHQ-2 asks participants about the frequency of depression symptoms. Based on PHQ-2 scores, participants are categorized into moderate, moderately severe, and severe symptoms (≥3) or no/mild depressive symptoms (<3). The score ranges from 0 to 6 with a score of 3 or more indicating a probable case of depression.

### Differences in cardiovascular health between US immigrants and non-immigrants

**Fig. 1:**
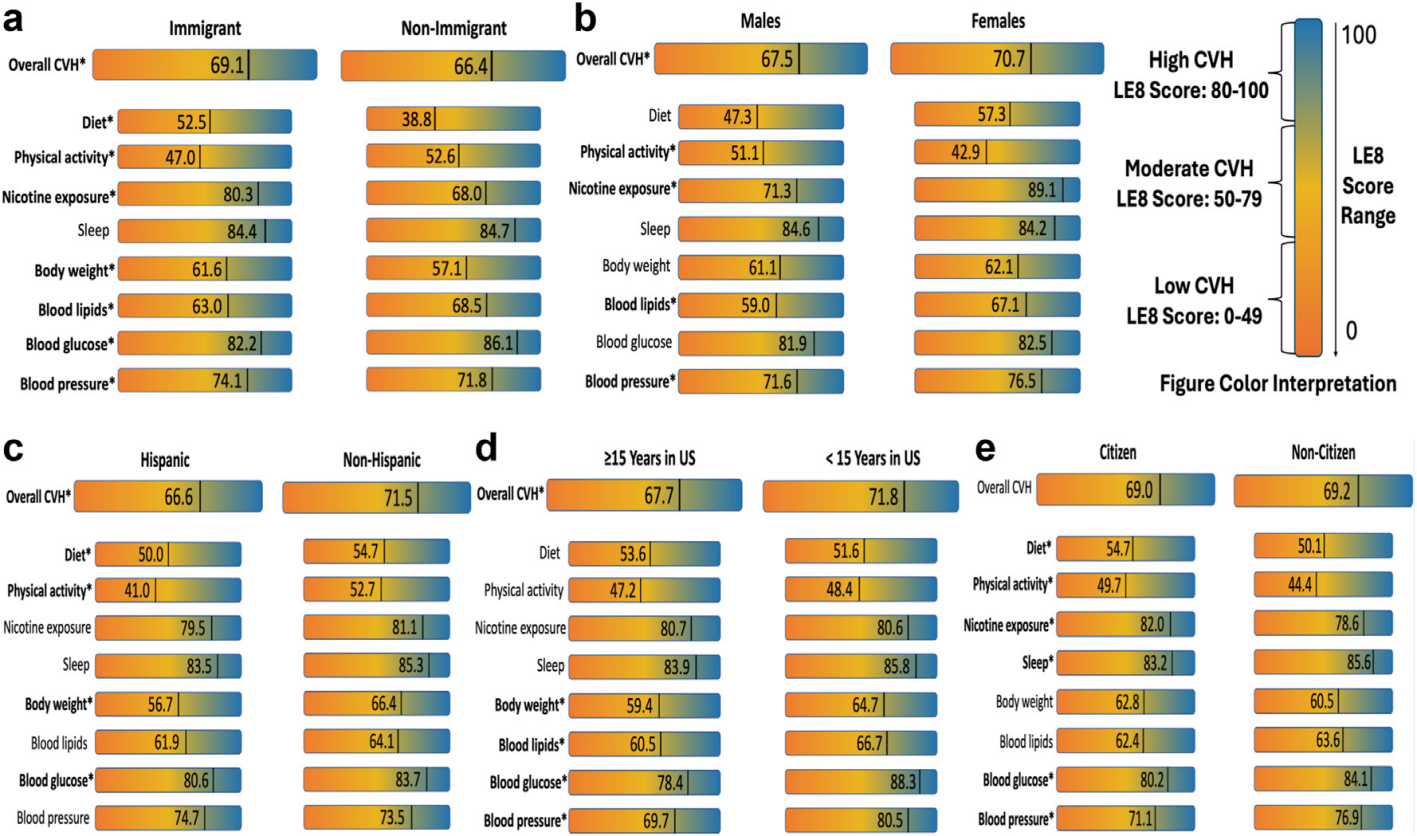
**Differences in cardiovascular health by nativity in the overall sample and by sex, ethnicity, acculturation, and citizenship among immigrants**. Figure 1 displays the mean overall Life's Essential 8 score and the individual metric scores (diet, physical activity, nicotine use, sleep health, body weight status, blood lipids, blood glucose, and blood pressure) among immigrants and non-immigrants (Panel a), and in the immigrant subpopulation by sex (Panel b), ethnicity (Panel c), acculturation (Panel d), and citizenship status (Panel e). Estimates in bold with ∗ indicate statistically significant differences (p-value < 0.05).

Differences in CVH between immigrants and non-immigrants are shown in Table 2 and Fig. 1 Panel a. For both groups, most participants had a LE8 score indicative of moderate CVH (64% for immigrants and 62% for US-born individuals). Immigrants had significantly higher prevalence of high CVH (26% vs. 23%) and lower prevalence of low CVH (10% vs. 15%) (p < 0.0001). In addition, they were significantly more likely to have higher scores for the diet (53 vs. 39, p < 0.0001), nicotine exposure (80 vs. 68, p < 0.0001), BMI (62 vs. 57, p < 0.0001), and blood pressure components (74 vs. 72, p = 0.006), but had lower scores for the physical activity (47 vs. 53, p < 0.0001), blood lipids (63 vs. 69, p < 0.0001), and blood glucose components (82 vs. 86, p < 0.0001). Notably, there were no significant differences in sleep health scores between immigrants and non-immigrants.

**Table 2:** Differences in cardiovascular health between United States immigrants and non-immigrants.^a^^,^^b^^,^^c^

	Immigrant	Non-immigrant	p-value
**CVH score categories prevalence % (N weighted)**			
High	26.4 (8,564,472)	23.3 (34,455,757)	<0.0001
Moderate	64.0 (20,781,269)	61.5 (90,781,746)	
Low	9.6 (3,126,354)	15.2 (22,458,849)	
**AHA Life's Essential 8 scores (out of 100 possible points) mean (SE)**			
Diet score	52.5 (1.13)	38.8 (1.03)	<0.0001
Physical activity score	47.0 (1.14)	52.6 (1.03)	<0.0001
Nicotine exposure score	80.3 (0.73)	68.0 (0.87)	<0.0001
Sleep health score	84.4 (0.56)	84.7 (0.41)	0.64
Body mass index score	61.6 (0.70)	57.1 (0.74)	<0.0001
Blood lipids (non-HDL cholesterol) score	63.0 (0.61)	68.5 (0.63)	<0.0001
Blood glucose score	82.2 (0.60)	86.1 (0.41)	<0.0001
Blood pressure score	74.1 (0.66)	71.8 (0.46)	0.006
Total LE8 score	69.1 (0.57)	66.4 (0.46)	<0.0001

CVH, Cardiovascular Health; LE8, Life's Essential 8.

a Independent samples t-tests were used to compare mean overall and individual Life's Essential 8 scores between immigrants and non-immigrants and Chi Square tests were used to compare the distribution of overall CVH between immigrants and non-immigrants.

b Cardiovascular health status was defined by the American Heart Association's Life's Essential 8 scores. LE8 scores of 80–100, 50–79, and 0–49 were considered indicative of high, moderate, and low CVH, respectively.

c All descriptive and analytical estimates consider NHANES analytical guidelines and sample weights to ensure that findings representative and generalizable to the broader United States population.

### Differences in cardiovascular health by sex, ethnicity, years in US, and citizenship status among US immigrants

Among immigrants, males had lower LE8 scores compared to females (68 vs. 71, p = 0.003) and had lower prevalence of high CVH (23% vs. 30%) (Table 3). When individual CVH metrics were evaluated, they also had lower diet (47 vs. 57, p < 0.0001), nicotine exposure (71 vs. 89, p < 0.0001), blood lipids (59 vs. 67, p < 0.0001), and blood pressure (72 vs. 77, p < 0.0001) component scores but higher physical activity scores (51 vs. 43, p < 0.0001) (Fig. 1, Panel b). Seep health, BMI, and blood glucose scores did not differ by sex. When LE8 scores were compared by ethnic groupings, Hispanic immigrants had a lower mean LE8 score compared to non-Hispanic immigrants (67 vs. 72, p < 0.0001), and only 1 in 5 had a LE8 score indicating high CVH compared to 1 in 3 among non-Hispanic immigrants (Table 3; Fig. 1, Panel c). Compared to non-Hispanic immigrants, Hispanic immigrants also had lower blood glucose (81 vs. 84, p = 0.001), BMI (57 vs. 66, p < 0.0001), physical activity (41 vs. 53, p < 0.0001), and diet scores (50 vs. 55, p = 0.004), but there were no differences in nicotine, sleep health, blood lipids, and blood pressure scores.

**Table 3:** Differences in Life's Essential 8 by sex, ethnicity, years in United States, and citizenship status among United States immigrants in the 2013–2018 National Health and Nutrition Examination Survey (N = 4503).^a^^,^^b^

	Males	Females	p-value
**Categories of CVH score prevalence % (N weighted)**			
High	22.5 (3,605,793)	30.1 (4,958,679)	0.004
Moderate	65.6 (10,496,799)	62.5 (10,284,470)	
Low	11.9 (1,904,558)	7.4 (1,221,796)	
**AHA Life's Essential 8 scores, mean (SE)**			
Diet score	47.3 (1.32)	57.3 (1.42)	<0.0001
Physical activity score	51.1 (1.57)	42.9 (1.16)	<0.0001
Nicotine exposure score	71.3 (1.07)	89.1 (0.81)	<0.0001
Sleep health score	84.6 (0.97)	84.2 (0.53)	0.73
Body mass index score	61.1 (1.05)	62.1 (1.01)	0.51
Blood lipids (non-HDL cholesterol) score	59.0 (0.76)	67.1 (0.98)	<0.0001
Blood glucose score	81.9 (0.79)	82.5 (0.69)	0.48
Blood pressure score	71.6 (0.86)	76.5 (0.78)	<0.0001
Total LE8 score	67.5 (0.82)	70.7 (0.70)	0.003

	Hispanic	Non-Hispanic	p-value
**Categories of CVH score prevalence % (N weighted)**			
High	19.8 (3,115,592)	32.6 (5,448,881)	<0.0001
Moderate	68.2 (10,743,945)	60.1 (10,037,324)	
Low	12.1 (1,904,889)	7.3 (1,221,465)	
**AHA Life's Essential 8 scores, mean (SE)**			
Diet score	50.0 (1.36)	54.7 (1.29)	0.004
Physical activity score	41.02 (1.20)	52.7 (1.56)	<0.0001
Nicotine exposure score	79.5 (0.90)	81.1 (1.32)	0.36
Sleep health score	83.5 (0.72)	85.3 (0.84)	0.09
Body mass index score	56.7 (0.80)	66.4 (0.84)	<0.0001
Blood lipids (non-HDL cholesterol) score	61.9 (0.68)	64.1 (1.11)	0.12
Blood glucose score	80.6 (0.69)	83.7 (0.76)	0.001
Blood pressure score	74.7 (0.87)	73.5 (0.94)	0.33
Total LE8 score	66.6 (0.57)	71.5 (0.71)	<0.0001

	≥15 years in United States	<15 years in United States	p-value
**Categories of CVH score prevalence % (N weighted)**			
High	23.2 (4,557,784)	32.8 (3,853,201)	<0.0001
Moderate	65.9 (12,927,079)	60.3 (7,092,521)	
Low	10.9 (2,141,617)	6.9 (813,027)	
**AHA Life's Essential 8 scores, mean (SE)**			
Diet score	53.6 (1.39)	51.6 (1.58)	0.27
Physical activity score	47.2 (1.45)	48.4 (1.66)	0.55
Nicotine exposure score	80.7 (0.86)	80.6 (1.27)	0.97
Sleep health score	83.9 (0.78)	85.8 (0.76)	0.05
Body mass index score	59.4 (0.73)	64.7 (1.04)	<0.0001
Blood lipids (non-HDL cholesterol) score	60.5 (0.84)	66.7 (1.05)	<0.0001
Blood glucose score	78.4 (0.73)	88.3 (0.81)	<0.0001
Blood pressure score	69.7 (0.71)	80.5 (0.83)	<0.0001
Total LE8 score	67.7 (0.62)	71.8 (0.82)	<0.0001

	Citizen	Non-Citizen	p-value
**Categories of CVH score prevalence % (N weighted)**			
High	10.4 (1,674,494)	8.9 (1,451,859)	0.25
Moderate	61.8 (9,975,871)	66.1 (10,805,398)	
Low	27.8 (4,483,954)	25.0 (4,080,518)	
**AHA Life's Essential 8 scores, mean (SE)**			
Diet score	54.7 (1.42)	50.1 (1.25)	0.003
Physical activity score	49.7 (1.44)	44.4 (1.47)	0.006
Nicotine exposure score	82.0 (1.17)	78.6 (0.87)	0.023
Sleep health score	83.2 (0.74)	85.6 (0.62)	0.004
Body mass index score	62.8 (0.95)	60.5 (0.95)	0.08
Blood lipids (non-HDL cholesterol) score	62.4 (0.85)	63.6 (0.69)	0.24
Blood glucose score	80.2 (0.72)	84.1 (0.85)	0.001
Blood pressure score	71.1 (0.87)	76.9 (0.87)	<0.0001
Total LE8 score	69.0 (0.81)	69.2 (0.61)	0.87

CVH, Cardiovascular Health; LE8, Life's Essential 8.

a Independent samples t-tests or Chi-square tests were used to compare mean LE8 scores and the distribution of CVH by sex (male vs. female), ethnicity (Hispanic vs. non-Hispanic), years living in the United States (≥15 years vs. <15 years), and citizenship status (United States citizen vs. non-citizen) within the United States immigrant population.

b All descriptive and analytical estimates consider NHANES analytical guidelines and sample weights to ensure that findings representative and generalizable to the broader United States population.

When CVH metrics were compared by years living in the US (proxy measure for acculturation), immigrants living in the US ≥15 vs. <15 years had a lower mean LE8 score (68 vs. 72, p < 0.0001) and lower prevalence of high CVH (23% vs. 33%, p < 0.0001) (Table 3; Fig. 1 Panel d). In addition, they had lower BMI (59 vs. 65, p < 0.0001), blood lipids (61 vs. 67, p < 0.0001), blood glucose (78 vs. 88, p < 0.0001), and blood pressure (70 vs. 81, p < 0.0001) scores with no significant differences observed for any of the LE8 health behavior scores. Finally, in analyses stratified by citizenship status, although there were no significant differences in overall CVH, immigrants who were US citizens had significantly higher diet (55 vs. 50, p = 0.003), physical activity (50 vs. 44, p = 0.006), and nicotine exposure scores (82 vs. 79, p = 0.023), but lower sleep health (83 vs. 86, p = 0.004) and blood glucose (80 vs. 84, p = 0.001), and blood pressure scores (71 vs. 77, p < 0.0001) with no differences observed in BMI and blood lipids scores (Fig. 1 Panel e).

### Association of sociodemographic factors, psychological health, and social determinants of health with cardiovascular health in US immigrants

**Fig. 2:**
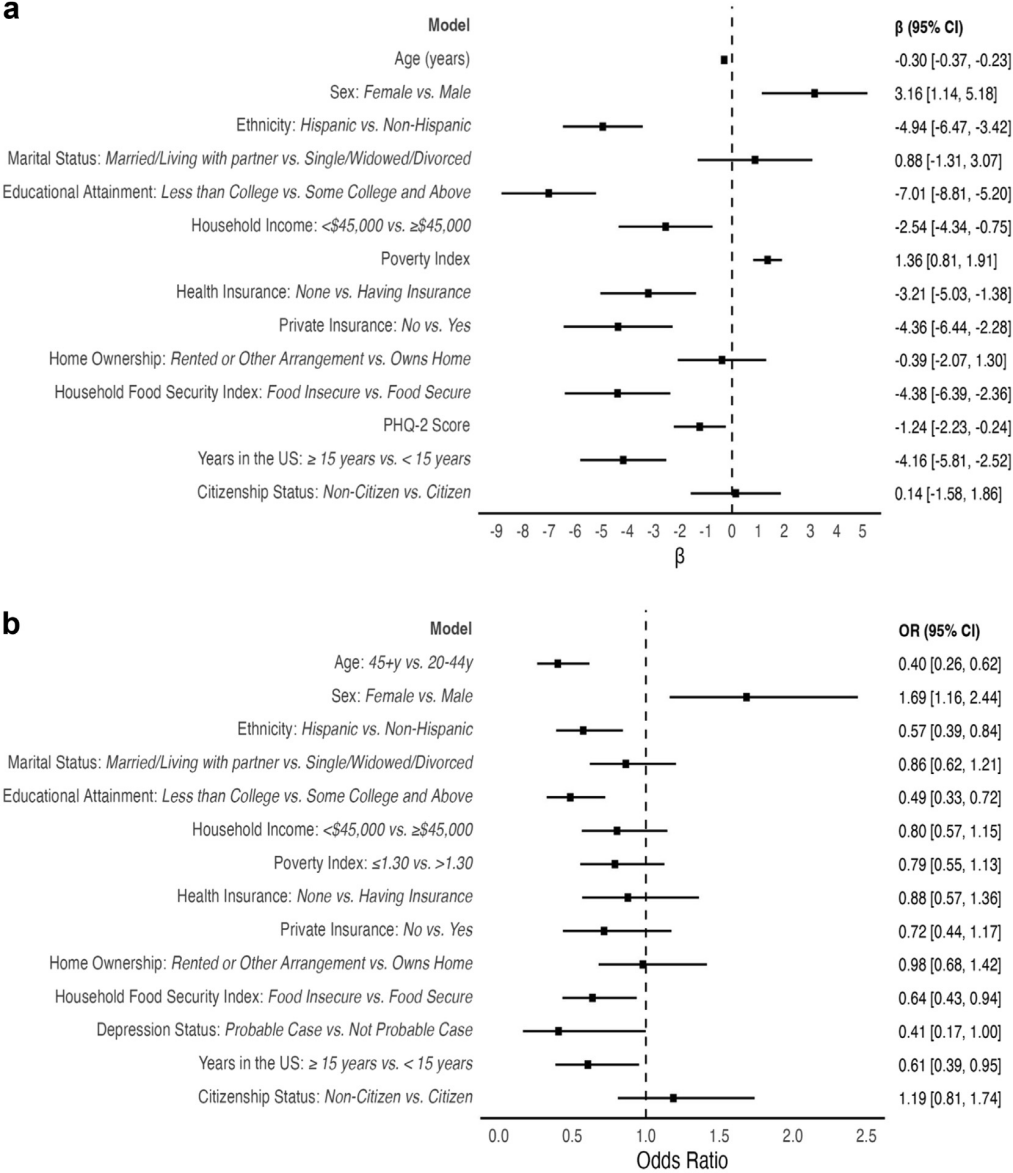
**Association of sociodemographic and socioeconomic characteristics with cardiovascular health in United States immigrants (N = 4503)**. Figure 2, Panel a displays results from survey-weighted linear models that evaluated sociodemographic and psychological health factors and social determinants of health in relation to the overall Life's Essential 8 score (LE8) (continuous). LE8 score was computed as the unweighted mean of the 8 component scores which range from 0 to 100 with higher LE8 scores representing better overall cardiovascular health (CVH). Results of linear regression models demonstrating positive beta coefficients indicate that the predictor variable is associated with higher LE8 scores (better CVH), while negative beta coefficients indicate that the predictor variable is associated with lower LE8 scores (lower cardiovascular health). All estimates consider NHANES analytical guidelines and sample weights to ensure that findings representative and generalizable to the broader United States (US) population. Panel b displays results from survey-weighted logistic models that evaluated sociodemographic and psychological health factors and social determinants of health in relation to odds of having moderate to high CVH defined by a LE8 score of 50–100. LE8 scores between 80 and 100 are indicative of high CVH, 50–79 moderate CVH, and 0–49 low CVH. Logistic regression results demonstrating odds ratios (ORs) <1 indicate that the predictor variable is associated with lower odds of having moderate to high CVH, while ORs >1 indicate that the predictor variable is associated with higher odds of having moderate to high CVH. All estimates consider NHANES analytical guidelines and sample weights to ensure that findings representative and generalizable to the broader US population. In linear regression models, age (per 1 year increment), Poverty Index, and Patient Health Questionnaire-2 (PHQ-2) score were modeled on the continuous scale. In logistic regression models all independent variables were dichotomized. Poverty Index was computed as the ratio of self-reported family income to poverty thresholds specific to family size defined by the Department of Health and Human Services. The poverty index ratio ranges from 0 to any number greater than 1. Below 1 signifies the family's income is below the poverty threshold, equal to 1 the family's income is exactly at the poverty threshold, and greater than 1 the family's income is above the poverty threshold. The poverty index was grouped into two categories: >1.30 and ≤1.30 consistent with commonly used percentages of the poverty guidelines (i.e., 130% of the guidelines), by federal programs in determining eligibility. Household Food Security Index was measured using the 18-item US Household Food Security Survey Module. The household food security status was computed from such that scores ranged from 0 to 18 and those with scores of ≥3 considered food insecure based on United States Department of Agriculture guidelines. Depression status was assessed using PHQ-2 which asks participants about the frequency of depression symptoms with scores ranging from 0 to 6 and a score ≥3 indicating a probable case of depression.

In the linear regression models, being female (β = 3.2, 95% CI: 1.1, 5.2) and a greater poverty index indicating higher household income (β = 1.4, 95% CI: 0.8, 1.9) were significantly associated with having a higher overall LE8 score in the immigrant population. In contrast, older age (β = −3.0, 95% CI: −3.7, −2.3 per 10 year increment in age), Hispanic ethnicity (β = −4.9, 95% CI: −6.5, −3.4), having an educational attainment less than college (β = −7.0, 95% CI: −8.8, −5.2), having household income <$45,000 (β = −2.5, 95% CI: −4.3, −0.8), lacking health insurance generally (β = −3.2, 95% CI: −5.0, −1.4), lacking private insurance specifically (β = −4.4, 95% CI: −6.4, −2.3), living in a food-insecure household (β = −4.4, 95% CI: −6.4, −2.4), poorer psychological health (β = −1.2, 95% CI: −2.2, −0.2), and living in the US for ≥15 years (β = −4.2, 95% CI: −5.8, −2.5) were all significantly associated with having a lower overall LE8 score in this population (Fig. 2; Panel a). Null results were observed for marital status, home ownership, and citizenship status. In exploratory analyses comparing Hispanic immigrants to Asian immigrants, Hispanic immigrants had significantly lower LE8 scores (β = −5.0, 95% CI: −6.4, −3.5).

In the logistic regression models, age ≥45 years (OR: 0.4, 95% CI: 0.3, 0.6), Hispanic ethnicity (OR: 0.6, 95% CI: 0.4, 0.8), having educational attainment less than college (OR: 0.5, 95% CI: 0.3, 0.7), living in a food-insecure household (OR: 0.6, 95% CI: 0.4, 0.9), having depression (OR: 0.4, 95% CI: 0.2, 1.0), and living ≥15 years in the US (OR: 0.6, 95% CI: 0.4, 0.95) were significantly associated with lower odds of having moderate to high CVH among US immigrants (Fig. 2, Panel b). The strongest effect sizes were observed for age ≥45 y, education less than college, and depression. In exploratory analyses, compared to Asian immigrants, Hispanic immigrants had 45% lower odds of having moderate to high CVH (OR: 0.55, 95% CI: 0.38–0.78). In contrast, being female was associated with 69% higher odds of having moderate to high CVH. Marital status, household income, poverty index, health insurance status, home ownership, and citizenship status were not significantly associated with odds of having moderate to high CVH in logistic models.

## Discussion

This investigation of CVH status using the enhanced LE8 framework in the US immigrant population demonstrates notable differences in overall CVH and its individual metrics between US immigrant and non-immigrant adults. Understanding the status of CVH and how critical contexts shape CVH in the diverse US immigrant population is instrumental for informing public health and policy initiatives aimed at mitigating health inequities. Immigrants had higher overall CVH, diet, nicotine exposure, BMI, and blood pressure scores, but lower physical activity, glucose, and cholesterol scores compared to US-born adults. The only CVH metric that did not vary among the two groups was the novel eighth sleep health metric. Among immigrant sub-populations, overall CVH was lower among those who were male, self-identified as Hispanic, and those who lived in the US for ≥15 years but did not differ by citizenship status, though results varied for individual CVH metrics. Lower education levels, not having health insurance, particularly private insurance, greater food insecurity, and poorer psychological health were associated with lower CVH among immigrants. Specifically, having less than college educational attainment, living in a food-insecure household, and having depression were associated with lower odds of having moderate to high CVH in this population.

Our results are aligned with previous studies that evaluated CVD risk in the US immigrant population.^1^^,^^5^, ^6^, ^7^^,^^12^^,^^20^, ^21^, ^22^, ^23^, ^24^, ^25^ In the Multi-Ethnic Study of Atherosclerosis, US-born participants had 37% lower odds (OR: 0.63) of optimal CVH, measured by the AHA's Life's Simple 7, compared with foreign-born participants.^25^ In a prior analysis of 2011–2016 NHANES data, immigrants vs. non-immigrants had a significantly lower prevalence of hypertension (29% vs. 35%) but a higher prevalence of diabetes (16% vs. 13%)^12^; this is consistent with our observation of higher blood pressure scores but lower glucose scores is in this population. Compared to findings from the AHA LE8 Presidential Advisory's accompanying publication regarding the status of CVH in the general US population using NHANES data, our findings show that immigrants generally have a higher prevalence of high CVH (26% vs. 20%) and lower prevalence of low CVH (10% vs. 18%) than the general population.^16^ Similarly, the mean CVH scores for males and females in the general population are lower than those observed for the immigrant population (62.5 vs. 67.5 and 67.0 vs. 70.7, respectively).^16^ This is consistent with prior work, which shows that despite being more socioeconomically disadvantaged and facing structural and institutional inequities in the host society, immigrants are paradoxically often shown to be healthier than the host population.^1^^,^^26^

In our study, Hispanic immigrants, in particular, had worse overall CVH compared to non-Hispanic immigrants, including Asian immigrants who represented the second largest immigrant group. This could be explained by the lower access to healthcare and lower treatment rates for cardiometabolic conditions in this population, as well as the higher prevalence of food insecurity and disparate poverty burden, particularly among Hispanic adults who are non-citizens.^1^^,^^2^^,^^4^^,^^12^ Importantly, Hispanic adults have been shown to have some of the highest prevalence rates of obesity and diabetes in the US (males 45.2%, females 45.7% and males 14.5%, females 12.3% respectively),^27^ and the prevalence of a low risk cardiometabolic profile characterized by favorable blood pressure, cholesterol, BMI, and absence of a diabetes diagnosis is less than 10% in this population.^11^ This is exacerbated by being a non-citizen, which has been linked to 75% higher odds of having uncontrolled blood glucose (A1C ≥8.0).^20^

Prior research also shows that, among immigrants, a longer duration in the US leads to gradual loss of the immigrant health advantage due to assimilation of unhealthy lifestyles and longer exposure to institutional and societal inequities, which could contribute to the deterioration of CVH status over time.^1^ Indeed, when the CVH status of immigrants was stratified by years lived in the US in our study, those who had been living in the US longer had CVH scores that are more similar to what has been previously reported for the general population.^16^ This may be reflective of the cumulative consequences of sustained structural racism and discrimination on immigrant populations, which may result in an increased burden of SDOH^28^^,^^29^; immigrants are more likely to experience poverty, live in under resourced communities, have limited access to preventive and critical health care, and be persistently exposed to anti-immigrant bias and rhetoric.^1^^,^^28^^,^^29^ In addition, acculturation, measured in different studies by proxies such as English proficiency, language spoken at home, or time in the US, etc. has been linked to lower CVH in other samples, reflected by higher rates of smoking, poorer diet and sleep, and higher BMI, which collectively impact risk for cardiometabolic outcomes such as diabetes, hypercholesterolemia, and obesity.^21^, ^22^, ^23^, ^24^ For instance, analyses from the Hispanic Community Health Study/Study of Latinos found that lower acculturation was significantly associated with up to 2-fold higher odds of having a low risk profile characterized by favorable blood pressure, BMI, and cholesterol and absence of diabetes, particularly among female participants.^11^ Similarly in the Multi-Ethnic Study of Atherosclerosis, foreign-born participants who lived the longest in the US had 38% lower odds of having optimal CVH, defined using the AHA's Life's Simple 7 framework, when compared to foreign-born participants who lived the shortest in the US.^25^

Notably, we did not observe significant differences for the new sleep health metric when comparing immigrants to US-born individuals. This is contrary to prior research, which demonstrates that immigration status exacerbates racial and ethnic sleep health disparities, particularly given that immigrants have a disparate burden of the SDOH that predispose to poorer sleep.^22^^,^^30^^,^^31^ It is possible that the null results for sleep are explained by the fact that the new sleep health metric is only defined by sleep duration. Sleep duration may not be a sufficient proxy of sleep health,^32^, ^33^, ^34^, ^35^ as prior studies show a higher prevalence of suboptimal sleep quality, sleep complaints, and sleep disorders in this population.^22^^,^^36^^,^^37^ In addition, it is possible that sleep health is shaped by changes in legal immigration status and policies related to immigration, a nuance that could not be captured with NHANES data. In fact, a recent study reported evidence of a significant improvement in immigrants' sleep duration in response to the approval of the Deferred Action for Childhood Arrivals (DACA) program in 2012 with benefits disappearing after 2016 and being undermined by uncertainty about the future.^31^ Thus, it is possible that the lack difference in sleep duration between immigrants and non-immigrants in this analysis is due, at least in part, to less hostile immigration policies during most of the study's timeframe.

The present study represents one of the earliest investigations of CVH and its determinants using the enhanced LE8 framework among US immigrants. Some strengths of the study include leveraging a nationally representative cohort for our analysis, the rigorous data collection procedures and gold standard approaches for assessing biomarkers in NHANES, use of the enhanced LE8 framework for assessing CVH, and investigation of CVH across key SDOH in this population. Study limitations include the cross-sectional nature of NHANES, which prohibits the evaluation of changes in CVH across the lifecourse in the immigrant population and how changes in SDOH and immigrant experiences over time shape CVH inequities. The reliance on self-report for measuring health behaviors and social and psychological factors may have resulted in recall bias and measurement error. For some variables with missingness rates >10% (e.g., having private insurance or poverty index), our analyses may have underestimated associations due to lower statistical power. In addition, the sample size, data limitations on self-reported race, ethnicity, and country of origin, and root causes of immigration prohibited the ability to examine differences in CVH metrics across ethnic subgroups, including the heterogenous Hispanic, Asian, and Middle Eastern and North African immigrant populations. The latter has been added as a new race and ethnicity category in the new Census categorization, which will enable investigation of CVH using disaggregated data in future research.^38^ Related to this, NHANES does not distinguish between noncitizens with or without documentation; immigrants may overreport citizenship (resulting in underestimation of results by citizenship status), and we also cannot rule out the possibility that undocumented migrants are not adequately represented. At the same time, lower survey response rates among immigrants due to younger age, longer work hours, and greater burden of adverse SDOH may have impacted the generalizability of these findings.^39^ Importantly, increasingly more immigrants, especially those who are citizens, are arriving as refugees and may have CVD risk factors not captured in NHANES such as exposure to traumatic events or displacement-related factors such as access to care along the migratory route.

While US immigrants have more favorable overall CVH compared to US-born persons, CVH status is complex and varies by CVH metric, including across immigrant sub-populations. Our findings highlight that glycemic control, physical inactivity, and blood lipids may be important targets for CVD prevention in the immigrant population, and that Hispanic immigrants and immigrants with more years in the US are key subpopulations to engage in CVH promotion efforts. Given that greater years lived in the US could adversely impact CVH among immigrants, interventions aimed at preserving favorable CVH components and healthy lifestyle habits among new immigrants could be impactful for preventing CVD in this population. In addition, policies that address socioeconomic inequities and mental health for the immigrant population at large could also have great impact. However, additional research is necessary to better understand immigration as a SDOH in the heterogonous US immigrant population and to capture nuanced differences in CVH and its cultural, societal, and institutional determinants for informing culturally responsive effective primordial prevention interventions. In addition, with rapidly evolving migration patterns and a growing number of individuals affected by forced displacement due to climate change, political conflict, and violence, the influence of the root causes driving immigration on CVH warrants investigation.^26^

## Contributors

NM participated in the design and concept of the study. All authors had access to the study data, and NM, RH, and VD accessed and verified the data in the study and take responsibility for the integrity of the data and the accuracy of the data analysis. RH conducted statistical analyses. All authors contributed to the acquisition, analysis, and interpretation of the data. NM and AL wrote the first and revised drafts of the manuscript. All authors contributed to the critical revision of the manuscript. NM obtained funding and provided supervision. All authors approved the final version of the manuscript for publication. NM was responsible for the decision to submit the manuscript for publication.

## Data sharing statement

NHANES data and guidance on analytical approaches are publicly and freely available from the US Centers for Disease Control and Prevention's National Center for Health Statistics and can be accessed at: https://www.cdc.gov/nchs/nhanes/index.htm.

## Declaration of interests

The authors have no conflicts of interest relevant to this article to disclose.
