# Supplementary material for: Status of cardiovascular health in United States immigrants using the American Heart Association's Life's Essential 8 framework: a population-based study of prevalence estimates and determinants
Source: Lancet Reg Health Am. 2025 May 13;46:101107. doi: 10.1016/j.lana.2025.101107 (PMC12141098; doi:10.1016/j.lana.2025.101107)
Supplement: Supplementary Figure and Table [file mmc1.pdf]

Status of Cardiovascular Health in United States Immigrants Using the American Heart Association's Life's Essential 8 Framework: A Population-Based Study of Prevalence Estimates and Determinants

Table of Contents

Supplementary Figure 1 3

Supplementary Table 1 4

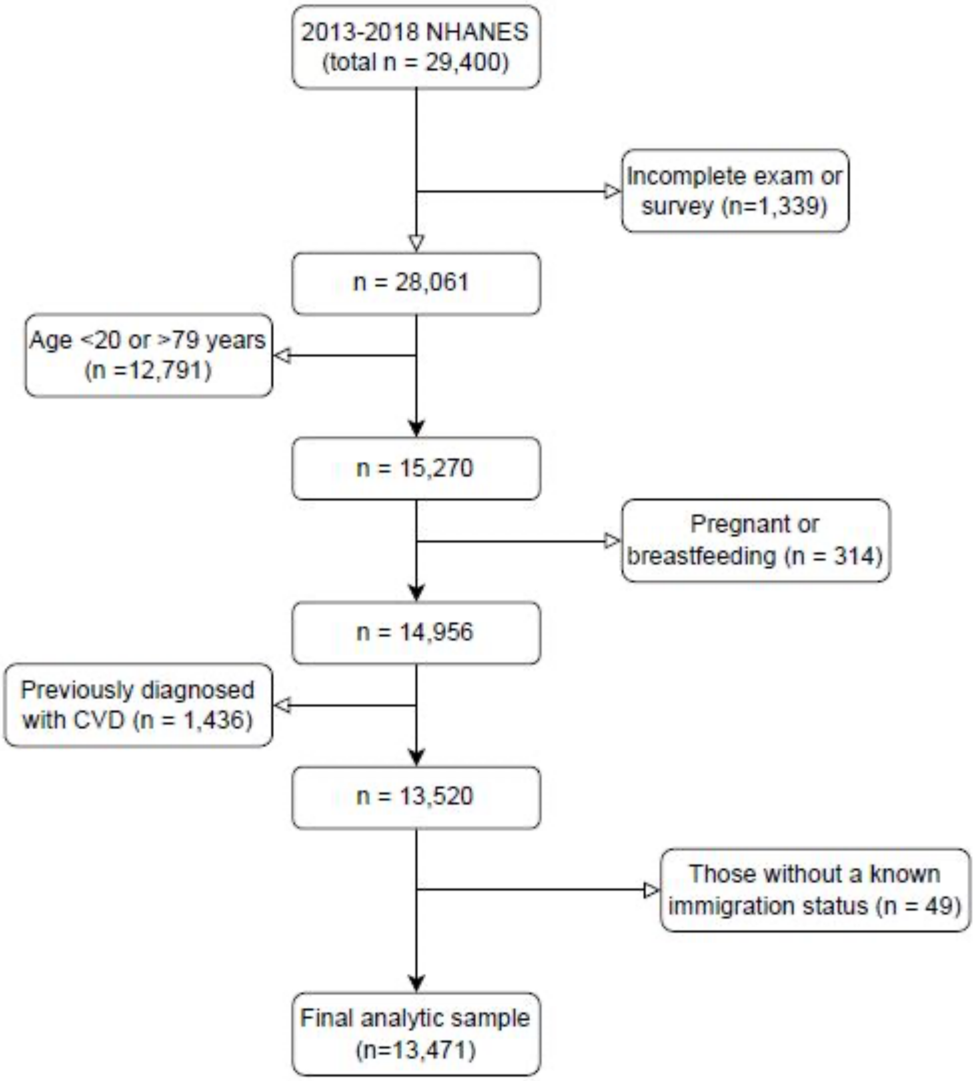

Supplementary Figure 1. Flow chart of analytical sample creation. NHANES, National Health and Nutrition Examination Survey; CVD, Cardiovascular disease.

Supplementary Figure Legend: The flowchart demonstrates how the analytical sample was created from the 2013-2018 NHANES datasets comprised of a total of 29,400 participants after application of the study eligibility criteria.

Supplementary Table 1. Scoring Approach for Quantifying the American Heart Association's Life's Essential 8 Cardiovascular Health Metrics [a,b]

Health Behaviors	Quantification of Metric	Health Factors	Quantification of Metric
**Diet**	Points — Quantiles of DASH-style Eating Pattern Adherence<br>100 — ≥95th percentile (top/ideal diet)<br>80 — 75th–94th percentile<br>50 — 50th–74th percentile<br>25 — 25th–49th percentile<br>0 — 1st–24th percentile (bottom/least ideal quartile)	**Body Mass Index**	Points — BMI (kg/m^2)<br>100 — <25<br>70 — 25.0-29.9<br>30 — 30.0-34.9<br>15 — 35.0-39.9<br>0 — ≥40.0<br><br>Scoring for those of Asian or Pacific Islander descent:<br>Points — BMI (kg/m^2)<br>100 — 18.5–22.9<br>75 — 23.0–24.9<br>50 — 25.0–29.9<br>25 — 30.0–34.9<br>0 — ≥35.0
**Physical Activity**	Points — Minutes/Week of moderate to vigorous intensity physical activity<br>100 — ≥150<br>90 — 120-149<br>80 — 90-119<br>60 — 60-89<br>40 — 30-59<br>20 — 1-29<br>0 — 0	**Blood Lipids**	Points — Non–HDL cholesterol (mg/dL)<br>100 — <130<br>60 — 130-159<br>40 — 160-189<br>20 — 190-219<br>0 — ≥220<br><br>If drug-treated level, subtract 20 points
**Nicotine Exposure**	Points — Use of cigarettes, inhaled NDS, and secondhand smoke exposure in the home<br>100 — Never smoker<br>75 — Former smoker, quit ≥5 y<br>50 — Former smoker, quit 1-<5 y<br>25 — Former smoker, quit <1 y, or currently using inhaled NDS<br>0 — Current smoker<br><br>Subtract 20 points (unless score is 0) if living with smoker	**Blood Glucose**	Points — Status<br>100 — No history of diabetes and (FBG < 100 or HbA1c <5.7)<br>60 — No diabetes and (FBG 100 – 125 or HbA1c 5.7–6.4) (prediabetes)<br>40 — Diabetes with HbA1c <7.0<br>30 — Diabetes with HbA1c 7.0–7.9<br>20 — Diabetes with HbA1c 8.0–8.9<br>10 — Diabetes with HbA1c 9.0–9.9<br>0 — Diabetes with HbA1c ≥10.0

Sleep Health

Points	Average Sleep Duration (Hours/Night)
100	7-<9
90	9-<10
70	6-<7
40	5-<6 or ≥10
20	4-<5
0	<4

Blood Pressure

Points	BP Level (mmHg)
100	<120/<80 (optimal)
75	120–129/<80 (elevated)
50	130–139 or 80–89 (stage 1 hypertension)
25	140–159 or 90–99
0	≥160 or ≥100

Subtract 20 points if treated level

[a]BMI, body mass index; BP, blood pressure; CVH, cardiovascular health; DASH, Dietary Approaches to Stop Hypertension; HbA1c, hemoglobin A1c; HDL, high-density lipoprotein; NDS, nicotine-delivery system; NHANES, National Health and Nutrition Examination Survey.

[b]CVH scoring algorithm based on approach described in: Lloyd-Jones DM, Allen NB, Anderson CAM, et al. Life’s Essential 8: updating and enhancing the American Heart Association’s construct of cardiovascular health: A Presidential Advisory from the American Heart Association. Circulation. 2022;146(5):e18–e43 and Lloyd-Jones DM, Ning H, Labarthe D, et al. Status of cardiovascular health in US adults and children using the American Heart Association’s new “Life’s Essential 8” metrics: prevalence estimates from the National Health and Nutrition Examination Survey (NHANES), 2013 through 2018. Circulation. 2022;146(11):822–835.
